# Identification and Characterization of Reference Genes for Normalizing Expression Data from Red Swamp Crawfish *Procambarus clarkii*

**DOI:** 10.3390/ijms160921591

**Published:** 2015-09-08

**Authors:** Hucheng Jiang, Zhaojun Qian, Wei Lu, Huaiyu Ding, Hongwei Yu, Hui Wang, Jiale Li

**Affiliations:** 1 Key Laboratory of Freshwater Fishery Germplasm Resources, Ministry of Agriculture, Shanghai Ocean University, Shanghai 201306, China; E-Mails: hcjiang@163.com (H.J.); zhaojunqian1216@163.com (Z.Q.); 2 Jiangsu Xuyi Riverred Crawfish Eco-Park Co., Ltd., Xuyi 211700, China; E-Mails: 15851705385@163.com (W.L.); yuhongwei-yhw@163.com (H.Y.); 3 Jiangsu Key Laboratory for Eco-Agricultural Biotechnoology Around Hongze Lake, Huaiyin Normal University, Huaian 223300, China; E-Mails: huaiyu-ding@163.com (H.D.); hytcwhh@hytc.edu.cn (H.W.)

**Keywords:** *Procambarus clarkii*, developmental stage, qRT-PCR, reference gene, normalization

## Abstract

qRT-PCR is a widely used technique for rapid and accurate quantification of gene expression data. The use of reference genes for normalization of the expression levels is crucial for accuracy. Several studies have shown that there is no perfect reference gene that is appropriate for use in all experimental conditions, and research on suitable reference genes in red swamp crawfish (*Procambarus clarkii*) is particularly scarce. In this study, eight commonly used crustacean reference genes were chosen from *P. clarkii* transcriptome data and investigated as potential candidates for normalization of qRT-PCR data. Expression of these genes under different experimental conditions was examined by qRT-PCR, and the stability of their expression was evaluated using three commonly used statistical algorithms, geNorm, NormFinder and BestKeeper. A final comprehensive ranking determined that *EIF* and *18S* were the optimal reference genes for expression data from different tissues, while *TBP* and *EIF* were optimal for expression data from different ovarian developmental stages. To our knowledge, this is the first systematic analysis of reference genes for normalization of qRT-PCR data in *P. clarkii*. These results will facilitate more accurate and reliable expression studies of this and other crustacean species.

## 1. Introduction

The red swamp crawfish *Procambarus clarkii* is a freshwater species native to the South-Central United States and Northeastern Mexico but has spread in range to become one of the most invasive species worldwide [1,2,3]. *P. clarkii* was introduced from Japan to Nanjing, China in 1929 [4,5], and this species has been farmed extensively in China since the 1990s, which is now the world’s leading crawfish producer [6,7].

The ovary is a component of both reproductive and endocrine systems and ensures oogenesis proceeds smoothly [8]. In order to develop novel methods for controlling gonad maturation in crawfish to improve yields, it is critical to understand the molecular mechanisms of ovarian development and oogenesis [9]. Oogenesis is a complicated differentiation process regulated by a vast number of intra- and extra-ovarian factors [10,11,12].

Understanding gene expression is essential for elucidating the functional mechanisms underlying this complex process. Quantitative real-time PCR (qRT-PCR) is a useful technique for measuring gene expression due to its high sensitivity, accuracy, and reproducibility [13,14]. However, many factors can affect the accuracy of qRT-PCR, including the quality of the RNA, the efficiency of reverse transcription, the primer specificity and amplification efficiency, and the statistical analysis methods employed [15]. A suitable reference gene is needed for normalizing expression levels, and these should ideally be expressed at a stable rate across different species, in different tissues and under different experimental conditions. Selection of reference genes is therefore critical for ensuring the accuracy of qRT-PCR analysis, and numerous studies have demonstrated that this selection can have a significant impact on expression levels. Ideally, reference genes should not be heavily influenced by other factors [16].

The most commonly used reference genes in crustaceans are β-actin (*ACTB*), 18S ribosomal RNA (*18S*), glyceraldehyde-3-phosphate dehydrogenase (*GAPDH*) and elongation factor 1α (*EF-1α*) [17,18,19,20]. To date, few studies on suitable reference genes in *P. clarkii* have been reported, and the use of unstable reference genes may lead to inaccurate results and should be avoided [21]. Consequently, novel reference genes are much needed for normalizing qRT-PCR data in this species.

Our knowledge of reproduction-associated genes in crustaceans has improved thanks to the high-throughput sequencing of transcriptome libraries [22,23,24,25], and we now have a large reference gene resource from which to select candidates for validating gene expression levels. In this study, we selected several frequently used crustacean reference genes from the *P. clarkii* transcriptome library [26] and evaluated their stability in eight tissues and six ovarian developmental stages. Results were assessed using the geNorm, NormFinder and BestKeeper statistical algorithms. Furthermore, in order to illustrate the usefulness of the newly identified reference genes, we analyzed the expression of two genes; vitellogenin (*Vg*), an interesting gene associated with ovarian maturation and oogenesis, and proliferating cell nuclear antigen (*Pcna*). This study will facilitate more accurate and reliable expression studies in *P. clarkii* and other crustacean species.

## 2. Results

### 2.1. Primer Specificity and Amplification Efficiency

PCR amplifications using each primer pair were confirmed by the presence of a single peak in the melting curve analysis [27] and a specific band of the expected size in agarose gel electrophoresis. An ideal reaction reaches an efficiency value close to 1.0, representing a PCR efficiency of 100% [19,26,27,28]. The results showed that the primer efficiency of the eight reference genes ranged from 96.0% to 103.6%, and the correlation coefficient (*R*^2^) values ranged from 0.992 to 0.999 (Table 1).

**Table 1:** Primers and PCR amplification efficiencies.

Gene	Primer Sequence (5ʹ–3ʹ)	Length (bp)	PCR Efficiency (%)	Correlation Coefficient (*R*^2^)	Genbank Accession No.
*ACTB*	F: ATTGCAGACAGGATGCAGAA R: GAAAGGGAAGCCAAGATGG	125	98.5	0.998	KR135165
*GAPDH*	F: GCCCAGAACATCATCCCATCT R:CGTCATCCTCAGTGTAACCCAAG	235	96.0	0.997	AB094145
*EF-1α*	F: CCACAAAGGCAGGTGAAAAGG R: ATTGGGTGAACCAAGCAGGG	110	100.6	0.998	KR135166
*UB*	F: TCCAGCCTCTCCTGCCTT R: CCTTCCTTATCCTGAATCTTTGCC	172	103.6	0.997	KR135167
*TUB*	F: CCACTTCCCTCTCGTCACC R: AACAGCACGCCATGTACTTT	155	103.5	0.992	KR135168
*TBP*	F: AAGGAAATACGCTCGGATTG R: CTGGCTGTGAGTGAGGACAA	136	100.1	0.997	KR135169
*EIF*	F: GGAATAAGGGGACGAAGACC R: GCAAACACACGCTGGGAT	126	98.9	0.997	KR135170
*18S*	F: TCCGCATCACACTCACGT R: TGGAACCCTCTCCACAGG	165	100.6	0.997	KR135172
*Vg*	F: CCAGAAGACGCCACAAGAA R: CAGAAGGCATCAGCCAATC	170	99.4	0.999	KR135171
*Pcna*	F: AGAGGCGGACTGAAGAGG R: TTGATGGCATCCAGCACT	133	100.6	0.995	KR135173

### 2.2. Cycle Threshold (C_t_) Values of Reference Genes

**Figure 1:**
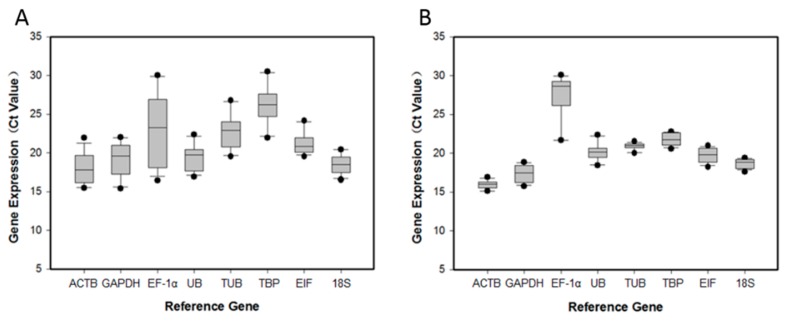
Expression levels of candidate reference genes under different experimental conditions. The expression levels of candidate reference genes in eight different tissues (**A**) and six different ovarian developmental stages (**B**) are displayed as cycle threshold (*C*_t_) values. The median is indicated by the line in the box. The interquartile range is bordered by the upper and lower edges, which indicate the 75th and 25th percentiles, respectively. The whiskers are inclusive of the maximal and minimal values, but exclusive of the outliers, which are represented by circles.

*C*_t_ values ranged from 15.11 to 30.48 overall, from 15.36 (*GAPDH*) to 30.48 (*TBP*) for samples in different tissues (Figure 1A) and from 15.11 (*ACTB*) to 30.04 (*EF-1α*) for samples in different ovarian developmental stages (Figure 1B). These results showed that *GAPDH* and *ACTB* were the most abundant transcripts with the lowest *C*_t_ values, whereas *TBP* and *EF-1α* had the lowest expression levels with the highest *C*_t_ values. Based on *C*_t_ dispersion, all candidate genes except *EF-1α* and *TBP* exhibited minimal variation.

### 2.3. Stability Analysis of Reference Genes

To further evaluate the stability of the candidate reference genes, the three commonly used algorithms geNorm, NormFinder and BestKeeper were applied to samples from different tissues and different ovarian developmental stages using the online integration tool RefFinder.

**Figure 2:**
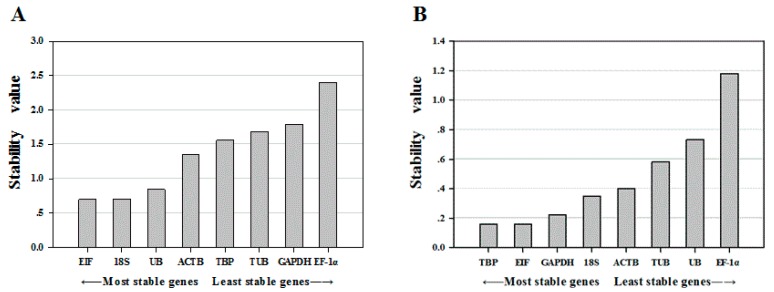
Expression stability of eight candidate reference genes calculated by geNorm. The expression stability value (M; *y*-axis) is a measure of gene expression stability. (**A**) Expression stability of different tissues in *P. clarkii*; (**B**) Expression stability of different ovarian developmental stages in *P. clarkii.*

The geNorm algorithm was used to calculate an expression stability value (M) where a lower value indicates more stable expression [28]. The results showed that among the different tissue samples, *EIF* and *18S* were the most stably expressed, while *GAPDH* and *EF-1α* were the least stably expressed (Table 2; Figure 2). Of the samples from the ovarian developmental stage, *TBP* and *EIF* were the most stably expressed, while *UB* and *EF-1α* were the least stably expressed. 

**Figure 3:**
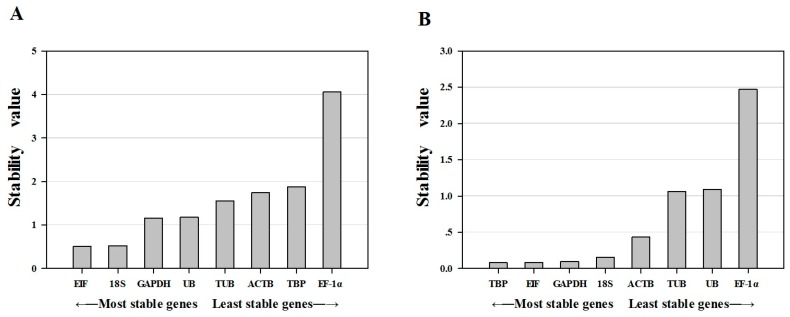
Expression stability of eight candidate reference genes calculated by NormFinder. The expression stability value (M; *y*-axis) is a measure of gene expression stability. (**A**) Expression stability of different tissues in *P. clarkii*; (**B**) Expression stability of different ovarian developmental stages in *P. clarkii.*

NormFinder was used to evaluate the stability of reference genes by calculating intra- and inter- group variation. A higher stability of expression is indicated by a lower average expression stability value [29]. The results showed that among the different tissue samples, *EIF* and *18S* were the most stably expressed, while *TBP* and *EF-1α* were the least stably expressed (Table 2; Figure 3). Of the samples from the ovarian developmental stage, *TBP* and *EIF* were the most stably expressed, while *UB* and *EF-1α* were the least stably expressed. The results of geNorm and NormFinder were therefore in good agreement.

BestKeeper uses the standard deviation (SD) and coefficient of variation (CV) of *C*_t_ values to evaluate expression stability, with more stable expression indicated by a lower SD [30]. Among the different tissues, *18S*, *EIF* and *UB* exhibited the lowest SD values and therefore the most stable expression, while *TBP* and *EF-1α* were the least stably expressed (Table 2 and Table 3). *TUB*, *ACTB* and *18S* exhibited the lowest SD values and the most stable expression of the ovarian developmental stage samples, while *UB* and *EF-1α* were the least stably expressed.

**Table 2:** Ranking of candidate reference genes under different experimental conditions.

	Rank	Method				
		geNorm	NormFinder	BestKeeper	Comprehensive Ranking
Different tissues	1	*EIF/18S*	*EIF*	*18S*	*EIF*
	2		*18S*	*EIF*	*18S*
	3	*UB*	*GAPDH*	*UB*	*UB*
	4	*ACTB*	*UB*	*TUB*	*GAPDH*
	5	*TBP*	*TUB*	*GAPDH*	*ACTB*
	6	*TUB*	*ACTB*	*ACTB*	*TUB*
	7	*GAPDH*	*TBP*	*TBP*	*TBP*
	8	*EF-1α*	*EF-1α*	*EF-1α*	*EF-1α*
Different ovarian developmental stages	1	*TBP/EIF*	*TBP/EIF*	*TUB*	*TBP*
	2			*ACTB*	*EIF*
	3	*GAPDH*	*GAPDH*	*18S*	*18S*
	4	*18S*	*18S*	*TBP*	*TUB/GAPDH*
	5	*ACTB*	*ACTB*	*EIF*	
	6	*TUB*	*TUB*	*GAPDH*	*ACTB*
	7	*UB*	*UB*	*UB*	*UB*
	8	*EF-1α*	*EF-1α*	*EF-1α*	*EF-1α*

Candidate reference genes were ordered from the most to the least stably expressed, based on their stability values calculated by different algorithms. The comprehensive rank for each gene is based on the geometric mean of its ranks from geNorm, NormFinder and BestKeeper. A lower geometric mean indicates higher stability.

**Figure 4:**
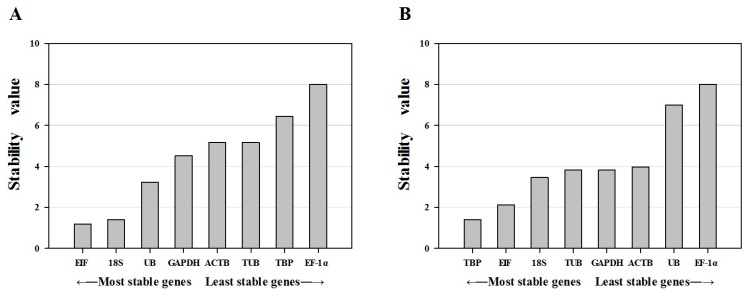
Expression stability of eight candidate reference genes calculated by RefFinder. (**A**) Expression stability of different tissues in *P. clarkii*; (**B**) Expression stability of different ovarian developmental stages in *P. clarkii.*

RefFinder is a comprehensive tool developed for evaluating reference genes and was used to confirm the results obtained from geNorm, NormFinder and BestKeeper. Based on the geometric mean obtained from several statistical approaches, we obtained the similar results for candidate reference genes. However, their ranks were slightly different under different experimental conditions (Table 2; Figure 4). Among the different tissue samples, the final comprehensive ranking suggests that the most stable reference genes were *EIF* and *18S*, followed by *UB*, *GAPDH*, *ACTB*, *TUB*, *TBP* and *EF-1α*. For the ovarian developmental stage samples, *TBP* and *EIF* were the most stably expressed genes, followed by *18S*, *TUB*, *GAPDH*, *ACTB*, *UB* and *EF-1α*. In all statistic algorithms, *EF-1α* was the least stably expressed among the eight tested genes. This gene should therefore not be used as a reference for normalization of qRT-PCR data in *P. clarkii*, certainly when comparing different tissues or ovarian developmental stage samples.

**Table 3:** Expression stability of candidate reference genes under different experimental conditions as calculated by BestKeeper.

	Statistical Parameter	Reference Gene							
		*ACTB*	*GAPDH*	*EF-1α*	*UB*	*TUB*	*TBP*	*EIF*	*18S*
Different tissues	GM [*C*_t_]	17.95	18.98	22.54	19.41	22.60	26.10	21.13	18.47
	AM [*C*_t_]	18.06	19.10	22.96	19.48	22.69	26.21	21.17	18.51
	Min [*C*_t_]	15.43	15.36	16.31	16.84	19.50	21.86	19.44	16.49
	Max [*C*_t_]	22.03	22.03	30.05	22.42	26.74	30.48	24.13	20.42
	SD [±*C*_t_]	1.87	1.86	3.71	1.38	1.71	1.96	1.08	0.94
	CV [%*C*_t_]	10.35	9.75	16.17	7.09	7.53	7.49	5.11	5.10
Different ovarian developmental stages	GM [*C*_t_]	15.96	17.33	27.21	20.14	20.89	21.74	19.70	18.65
	AM [*C*_t_]	15.97	17.36	27.37	20.18	20.90	21.75	19.72	18.66
	Min [*C*_t_]	15.11	15.73	21.65	18.41	20.03	20.53	18.24	17.63
	Max [*C*_t_]	16.94	18.84	30.04	22.32	21.52	22.78	20.97	19.41
	SD [±*C*_t_]	0.47	0.90	2.29	0.94	0.33	0.64	0.74	0.51
	CV [%*C*_t_]	2.95	5.18	8.38	4.66	1.56	2.95	3.77	2.74

### 2.4. Validation of the Usefulness of the Selected Reference Genes

It has been demonstrated that the use of unstable reference genes can dramatically increase the inaccuracy of expression data of target genes. In order to confirm the validity of the tested candidate reference genes, the relative expression level of the ovarian developmental-associated *Vg* and *Pcna* were normalized using the two most stably expressed and the least stably expressed genes as determined by RefFinder as described above.

**Figure 5:**
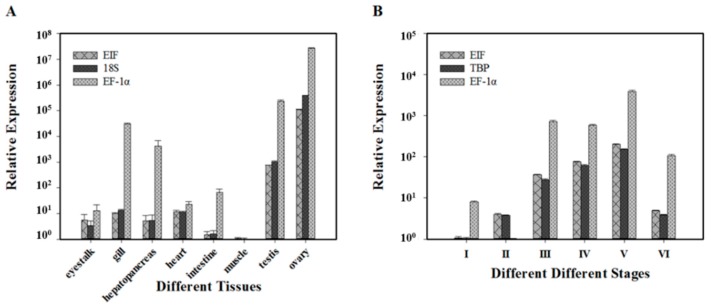
Expression levels of *Vg* using different candidate reference genes for normalization. (**A**) *Vg* expression patterns in eight different tissues. Different bars indicate *Vg* expression levels normalized against *EIF*, *18S* and *EF-1α*; (**B**) *Vg* expression patterns in six different ovarian developmental stages. Different bars indicate *Vg* expression levels normalized against *EIF*, *TBP* and *EF-1α*. No developmental stage = I, early developmental stage = II, previtellogenic stage = III, vitellogenic stage = IV, mature stage = V, post-spawning stage = VI.

Using the most stable reference genes (*EIF* and *18S*), the relative expression level of *Vg* was highest in gonad tissues, especially in ovary (Figure 5A). In contrast, *Vg* expression levels were higher in gill and hepatopancreas tissue when the least stable reference gene (*EF-1α*) was used for normalization. The relative expression level of *Vg* during different ovarian developmental stages provided insight into the function of *Vg* in this process (Figure 5B). *Vg* expression levels were significantly lower during no developmental stages (I) and during early development (stage II) when normalized against the most stable reference genes (*EIF* and *TBP*), and gradually increased to peak at the mature stage (V) before decreasing significantly at the post-spawning stage (VI). These results were similar to those reported previously for *Vg* in both *P. clarkii* [31] and *Litopenaeus vannamei* [32]. However, when the least stable reference gene (*EF-1α*) was used for normalization, *Vg* expression levels were higher at stages I and II, and also higher from the previtellogenic stage (III) to mature (V) stages.

**Figure 6:**
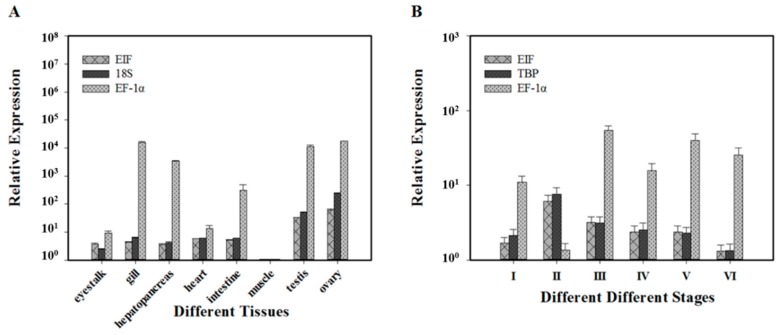
Expression levels of *Pcna* using different candidate reference genes for normalization. (**A**) *Pcna* expression patterns in eight different tissues. Different bars indicate *Vg* expression levels normalized against *EIF*, *18S* and *EF-1α*; (**B**) *Pcna* expression patterns in six different ovarian developmental stages. Different bars indicate *Vg* expression levels normalized against *EIF*, *TBP* and *EF-1α*. No developmental stage = I, early developmental stage = II, previtellogenic stage = III, vitellogenic stage = IV, mature stage = V, post-spawning stage = VI.

*Pcna* is an accessory protein of DNA polymerase δ and plays an essential role in nucleic acid metabolism [33]. Data showed that *Pcna* is expressed highly in proliferating tissues such as testis and ovary in shrimp [34]. In the present study, when the relative expression of *Pcna* was normalized against the most stable reference genes (*EIF* and *18S*), the data mirrored this trend with higher expression levels in testis and ovary. In contrast, normalization against the least stable reference gene (*EF-1α*) resulted in higher expression in gill, hepatopancreas and intestine tissue (Figure 6A). A previous study in the developing ovary of *Marsupenaeus japonicus* identified differential expression of *Pcna*, with lowest expression before development and highest expression during stage II [35]. In the present study, *Pcna* showed a similar expression profile, with lowest levels before development (stage I), peak expression during stage II, and a gradual decrease to the post-spawning stage (VI) when normalized against the most stable reference genes (*EIF* and *18S*) (Figure 6B). These results further suggest that *Pcna* plays an important role in ovarian development, especially in cell proliferation. However, the *Pcna* expression pattern differed significantly when normalized against the least stable reference gene (*EF-1α*), emphasizing the importance of the reference genes chosen for normalization.

## 3. Discussion

Analysis of gene expression under different experimental conditions is important for informative functional analysis, and qRT-PCR is one of the most widely used methods for quantifying gene expression [14,36]. However, since no potential reference genes are likely to be stably expressed under all possible experimental conditions, it is advisable to validate candidate reference genes under the required experimental conditions prior to their use in qRT-PCR normalization, rather than using previously published reference genes under different conditions [13,21,37].

Recent studies showed that many traditional reference genes are not stably expressed under different conditions [38,39,40,41,42], and the results of the present study support this. Frequently used reference genes such as *ACTB*, *UB* and *EF-1α* have been found to be inappropriate for normalization of expression data in *P. clarkii.* In the present work, *EF-1α* was the least stably expressed gene among different tissues and during different ovarian development stages, *UB* was ranked the next least stable, and *ACTB* expression was of intermediate stability.

Some previous studies have focused on identifying reference genes in other crustaceans [43,44], but research on *P. clarkii* is lacking. For accurate measurement of gene expression, reference genes should be stably expressed under given experimental conditions [37]. In the present study, eight candidates were tested among different tissues and during different ovarian developmental stages using the commonly used statistical algorithms geNorm, NormFinder and BestKeeper.

Furthermore, the results obtained from the different algorithms were relatively consistent in terms of the most and the least stably expressed genes. Slight differences in ranking between different programs was ascribed to their different statistical algorithms. *18S* and *EIF* were the most stably expressed among the different tissues tested. *EF-1α* was found to be the least stable reference gene by all the three statistical algorithms, and is not suitable for use in qRT-PCR analysis among different tissues from *P. clarkii*, despite being an ideal reference gene in ovarian tissue from *Peneaus monodon* [43]. Based on the final ranking, *EIF* and *18S* were designated as the most appropriate reference genes for normalization of gene expression in different tissues of *P. clarkii*. The results for different ovarian developmental stages were similar overall, but *TBP* and *EIF* were the most stable reference genes, while *EF-1α* was the least stable. Consequently, *TBP*, *EIF* and *18S* were considered to be the most appropriate reference genes for normalization of expression data during ovarian developmental stages of *P. clarkii*.

To investigate the effects of using different reference genes, the relative expression levels of the ovarian developmental-associated *Vg* and *Pcna* were investigated in different tissues and different ovarian developmental stages. Normalization using the most stable reference genes (*EIF* and *TBP*) were consistent with previous reports on *Vg* and *Pcna* expression in crustaceans, however normalization using the least stable reference gene (*EF-1α*) generated inconsistent results [31,32,45]. These results demonstrated that selection of different reference genes can have a significant impact on expression data, as previously reported [46].

## 4. Experimental Section

### 4.1. Crawfish Tissue Sample Collection

The handling of crawfish was conducted in accordance with the guidelines on the care and use of animals for scientific purposes set by the Institutional Animal Care and Use Committee (IACUC) of Shanghai Ocean University, Shanghai, China. The crawfish used in this project were obtained from Jiangsu Xuyi River red Crawfish Eco-Park, Jiangsu Province, China. Before tissue collection, crawfish were cultured in a tank of continuously aerated freshwater at ambient temperature (28 °C) for 72 h. Eyestalk, gill, hepatopancreas, heart, intestine, muscle, testis and ovary tissue were collected from six healthy, sexually mature crawfish. Based on a previous study [47], we classified the ovarian developmental cycle of *P. clarkii* into six stages: no developmental (I), early developmental (II), previtellogenic (III), vitellogenic (IV), mature (V) and post-spawning (VI). Ovarian developmental stage tissue (including the six stages described above) was surgically removed from six individuals per developmental stage, immediately frozen in liquid nitrogen, and stored at −80 °C until RNA extraction. Thus, eight tissue samples and six ovarian developmental stage samples were used in the subsequent analysis.

### 4.2. RNA Extraction and Reverse Transcription

Total RNA from each sample was isolated using TRIzol (Invitrogen, Carlsbad, CA, USA) following the manufacturer’s instructions, incubated with 10 U DNase I (Ambion, Austin, TX, USA) at 37 °C for 1 h and purified with a MicroPloy (A) Purist Kit (Ambion, Austin, TX, USA) according to the manufacturer’s instructions. Purified mRNA was dissolved in RNase/DNase-free ddH_2_O, and a NanoDrop (Thermo, Wilmington, DE, USA) was used to determine the sample quality and final concentration. The cDNA template for qRT-PCR was generated from 500 ng of total RNA with a PrimeScript RT reagent Kit (Takara, Dalian, China) in accordance with the manufacturer’s instructions. All cDNA samples were diluted to 50 ng/μL and stored at −20 °C for qRT-PCR analysis.

### 4.3. Reference Gene Selection and Primer Design 

From 22,652 assembled transcripts in the *P. clarkii* transcriptome library [26], we selected seven commonly used potential reference genes for expression stability analysis, namely β-actin (*ACTB*), elongation factor-1α (*EF-1α*), ubiquitin (*UB*), α-tubulin (*TUB*), TATA-binding protein (*TBP*), eukaryotic translation initiation factor 5A (*EIF*) and 18S ribosomal RNA (*18S*) (Table 4). Additionally, glyceraldehyde-3-phosphate dehydrogenase (*GAPDH*) was obtained from the NCBI database. The relative expression levels of *Vg* [48] and *Pcna* [35] were determined to confirm the validity of the tested candidate reference genes.

Sequences with high sequence similarity to candidate reference genes were aligned separately to highlight potential areas of polymorphism. Specific primers (Table 1) were designed using the Primer Premier 5.0 program [49], and primer pairs were selected from sequence regions with the fewest polymorphisms. The annealing temperature was 60 °C for all primer pairs, and the length of PCR products was 100–250 bp. PCR efficiency and correlation coefficients (*R*^2^) were determined based on the slopes of the standard curves generated using a 10-fold serial dilution of cDNA template. Primer specificity was confirmed by the presence of a single product of the expected size following 1.5% agarose gel electrophoresis and ethidium bromide staining. In addition, target amplicons were sequenced to confirm specificity of the PCR products.

**Table 4:** Candidate reference genes investigated in this study.

Abbreviation	Gene Name	Gene Function
*ACTB*	β-Actin	Cytoskeletal structural protein
*GAPDH*	Glyceraldehyde-3-phosphate Dehydrogenase	Oxidoreductase in glycolysis and gluconeogenesis
*EF-1α*	Elongation factor-1α	Protein biosynthesis
*UB*	Ubiquitin	Protein degradation
*TUB*	β-Tubulin	Cytoskeletal structural protein
*TBP*	TATA-binding protein	RNA polymerase transcription factor
*EIF*	Eukaryotic translation initiation factor 5A	Protein synthesis
*18S*	18S ribosomal RNA	Ribosome subunit

### 4.4. Quantitative Real-Time PCR 

Expression levels of the eight selected genes and the ovary-associated genes *Vg* and *Pcna* were measured using qRT-PCR performed in a 96-well plate with the CFX 96 real time system (Bio-Rad, Hercules, CA, USA). Each reaction of 25 μL contained 12.5 μL of 2× SYBR Green Real-Time PCR Master Mix, 2 μL of cDNA (100 ng), 9.5 μL of RNase/DNase-free ddH_2_O and 0.5 μL of each forward and reverse primer (10 mM). PCR conditions were 95 °C for 10 min, followed by 40 cycles of 95 °C for 10 s and 60 °C for 30 s, and generation of a dissociation curve by increasing the temperature from 65 to 95 °C in 0.5 °C increments to check for specificity of amplification. Each sample was amplified in triplicate (three technical replicates).

Baseline and threshold cycle (*C*_t_) values were automatically determined using Bio-Rad CFX manager version 1.6 with default parameters. The ΔΔ*C*_t_ method was used to evaluate the quantities of each amplified product according to the user manual. All figures were drawn with SigmaPlot 12.5 [50].

### 4.5. Analysis of Gene Expression Stability

The web-based tool RefFinder [51] (www.leonxie.com/referencegene.php) that integrates the algorithms geNorm [28], NormFinder [29] and BestKeeper [30] was used to evaluate the stability of the expression of the eight potential reference genes. For each gene, RefFinder estimated the geometric mean of the ranks calculated using the aforementioned approaches. Finally, we selected the ideal reference genes determined by RefFinder with lowest rank geometric mean. In order to confirm that the tested genes were suitable as internal controls for qRT-PCR analysis in *P. clarkii*, the two most stable and the least stable potential reference genes as determined by RefFinder were used to normalize the expression level of *Vg* in the same qRT-PCR conditions described above.

## 5. Conclusions

To the best of our knowledge, this is the first investigation into the expression stability of reference genes in *P. clarkii*. We identified and tested eight candidate reference genes for their ability to normalize qRT-PCR expression data from eight different tissues and six different ovarian developmental stages. These results will be useful for ensuring that future qRT-PCR data from this species and other crustaceans is accurate and reliable.
